# Towards standardization of the parameters for opening the blood–brain barrier with focused ultrasound to treat glioblastoma multiforme: A systematic review of the devices, animal models, and therapeutic compounds used in rodent tumor models

**DOI:** 10.3389/fonc.2022.1072780

**Published:** 2023-02-16

**Authors:** Rasika Thombre, Griffin Mess, Kelley M. Kempski Leadingham, Shivani Kapoor, Andrew Hersh, Molly Acord, Tarana Kaovasia, Nicholas Theodore, Betty Tyler, Amir Manbachi

**Affiliations:** ^1^ Department of Neurosurgery, Johns Hopkins University School of Medicine, Baltimore, MD, United States; ^2^ Department of Biomedical Engineering, Johns Hopkins University, Baltimore, MD, United States; ^3^ HEPIUS Innovation Laboratory, School of Medicine, Johns Hopkins University, Baltimore, MD, United States; ^4^ Department of Electrical Engineering and Computer Science, Johns Hopkins University, Baltimore, MD, United States; ^5^ Department of Mechanical Engineering, Johns Hopkins University, Baltimore, MD, United States; ^6^ Department of Anesthesiology, Johns Hopkins University School of Medicine, Baltimore, MD, United States

**Keywords:** blood-brain barrier, chemotherapy, focused ultrasound, glioblastoma multiforme, immunotherapy, microbubble, neuro-oncology, non-invasive

## Abstract

Glioblastoma multiforme (GBM) is a deadly and aggressive malignant brain cancer that is highly resistant to treatments. A particular challenge of treatment is caused by the blood–brain barrier (BBB), the relatively impermeable vasculature of the brain. The BBB prevents large molecules from entering the brain parenchyma. This protective characteristic of the BBB, however, also limits the delivery of therapeutic drugs for the treatment of brain tumors. To address this limitation, focused ultrasound (FUS) has been safely utilized to create transient openings in the BBB, allowing various high molecular weight drugs access to the brain. We performed a systematic review summarizing current research on treatment of GBMs using FUS-mediated BBB openings in *in vivo* mouse and rat models. The studies gathered here highlight how the treatment paradigm can allow for increased brain and tumor perfusion of drugs including chemotherapeutics, immunotherapeutics, gene therapeutics, nanoparticles, and more. Given the promising results detailed here, the aim of this review is to detail the commonly used parameters for FUS to open the BBB in rodent GBM models.

## 1 Introduction

In order to treat diseased lesions of the brain such as glioblastoma multiforme (GBM) and other brain cancers, therapeutic drugs must pass through the blood–brain barrier (BBB). However, the BBB is relatively impermeable, and its cellular composition creates a closed, homeostatic, intracranial environment separate from the rest of the body (1). The BBB is uniquely composed of multiple cell types that make it difficult for passive diffusion of molecules greater than 400 Da into the central nervous system (CNS), regardless of lipid solubility (1). The BBB additionally contains multiple types of active efflux transporter proteins that can pump out unwanted molecules from the CNS (1, 2). A brain tumor such as a GBM is generally considered “leaky” where the integrity of the BBB is compromised, increasing its permeability for larger compounds. This phenomenon results in bright areas denoted in magnetic resonance imaging (MRI) images from contrast agents like gadolinium (MAGNEVIST^®^, 938 Da). This enhanced permeability is thought to be heterogenous throughout the tumor, as BBB disruption is more prevalent in the core of the tumor while the outer tumor regions remain intact. This can also lead to uneven treatment distribution within a tumor (3, 4). The BBB creates clear design constraints on drug delivery for CNS tumors.

Due to the deadly and infiltrating geometry of GBM, there are many approaches being studied to develop improved therapeutic approaches. Chemotherapy is the current standard of care for most cancers and is often used in conjunction with other treatments. Many chemotherapeutics that are successful in treating other cancers [e.g., doxorubicin (DOX) and etoposide (ETO)] are limited in their ability to cross the BBB in therapeutic concentrations (5). Smaller chemotherapeutics that can diffuse through the BBB have other characteristics that slow their ability to accumulate in the brain, including quick plasma clearance and the presence of active drug efflux pumps (6). One example of this is temozolomide (TMZ), a commonly used lipophilic chemotherapeutic for GBM that has a molecular weight (MW) of 194 Da and that has shown significant increases in median survival of patients (7). Oral TMZ has demonstrated inefficient delivery, with only 20% of the drug in the blood plasma successfully accumulating in the brain parenchyma following oral delivery (8).

Immunotherapy, which can include delivering substances to either boost the immune system or help the body identify and kill cancer cells, is another treatment that is hindered by the BBB. Certain antibodies and other immunological macromolecules are too large (∼150 KDa) to diffuse through the BBB (9, 10). Researchers have been exploring the use of delivery vehicles such as nanoparticles to improve tissue targeting, pharmacokinetics, and prevent off-target cytotoxicity for immunotherapies, gene therapies and chemotherapies; however, tailored engineering of the molecular formations would be required to penetrate the BBB (11, 12).

Focused ultrasound (FUS) is a non-invasive therapeutic technology that has been investigated for the treatment of GBM. FUS uses ultrasound transducers to converge high-intensity ultrasound waves into a millimeter-sized focal region, where the combined beams can have a thermal or mechanical therapeutic effect at the tissue site (13). One application of FUS is the transient opening of the BBB, which can allow temporary access of therapeutics into the brain. When the focal region of the FUS beam is aimed toward intravenously-injected microbubbles, measuring just microns in diameter, the microbubbles will oscillate with the rarefactions and compressions of the applied pressure wave. This oscillation has sufficient energy to impart shear stresses onto the cells, which can break the bonds that keep the endothelial cells tightly linked. (14). There is also evidence that FUS applied to endothelial cells can increase active transport of molecules across the BBB *via* multiple pathways. Up-regulation of vesicles and carrier proteins has been observed following FUS exposure, enabling transcytosis of certain molecules from the blood vessels into the brain (15, 16). Additionally, shear stress generated by FUS is thought to have an effect on modulating mechanosensitive ion channels, which through various metabolic processes, can increase BBB permeability (16, 17). The length of opening of the BBB is parameter dependent; studies have shown that rapid, short ultrasound pulses can open the BBB for minutes with minimal energy required (18). Alternatively, higher amplitude FUS has been shown to induce BBB openings that have lasted more than 24 hours due to inertial cavitation, but there are saftey risks that are involved with inertial cavitation (19). Generally, FUS-induced BBB openings have shown the ability to significantly and safely increase drug perfusion (20). Importantly, multiple groundbreaking clinical trials have demonstrated both safety and feasibility in patients. (21) showed that repeated BBB openings were safe, (22) was able to show increased perfusion in brain tumors following FUS mediated BBB opening.

**Figure 1:**
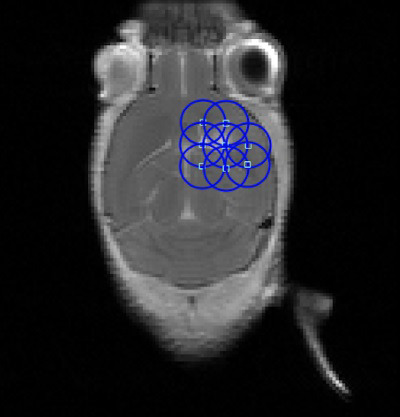
Example Focused Ultrasound (FUS) Sonication grid overlaid on an axial, contrast-enhanced T1-weighted magnetic resonance imaging (MRI) of a mouse with an orthotopically-implanted glioblastoma multiforme (GBM) tumor.

FUS-induced opening of the BBB is promising for the treatment of GBMs. Chemotherapies, immunotherapies, gene therapies, and radiotherapies have been tested with and without nanoparticles in conjunction with FUS-induced BBB openings *in vivo*, and some have translated into human clinical trials (23, 24). Many anti-cancer drugs have shown significant survival benefits and reduced tumor size when tested in intracranial glioma-bearing rodents after the application of FUS. FUS is a relatively new technique that has only gained momentum in the past few decades due to more recent technological advances, such as the incorporation of MR guidance and multielement transducers that can modulate the firing pattern of the FUS beam to correct for inaccurate beam propagation from the skull (**Figure 1**) (25). Utilizing its benefits to increase therapeutic drug delivery is currently being explored and the parameters being refined. FUS is highly dependent on the physical parameters of transducers, with many variables to optimize (e.g., FUS exposure time). While there have been review papers that discuss FUS and BBB permeabilization (26, 27), there are currently no standardized FUS treatment parameters. The goal of this systematic review is to analyze FUS treatment methods and parameters in rodent brain tumor models, and determine therapeutic indicators for optimal BBB permeabilization in the treatment of GBM. This review may serve as a reference for FUS-induced BBB permeability studies.

## 2 Methods

**Figure 2:**
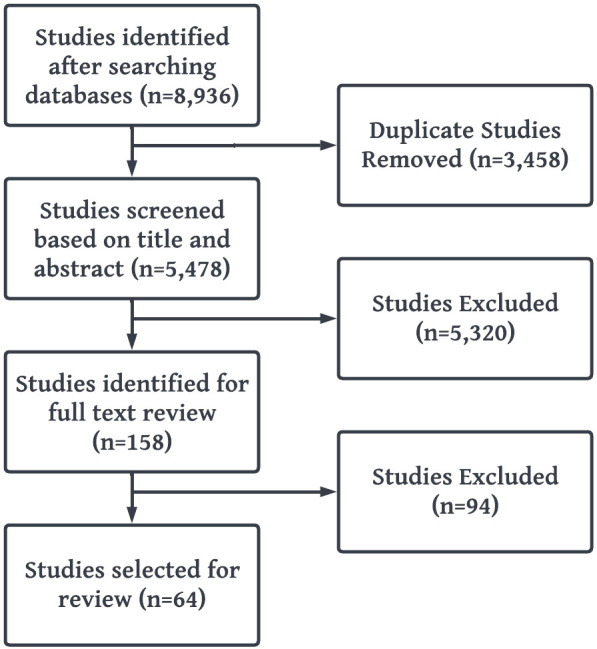
PRISMA flow diagram of study selection.

The Cochrane and Preferred Reporting Items for Systematic Reviews and Meta-Analyses (PRISMA) guidelines were followed for conducting and reporting the results of this study (**Figure 2**) (28).

### 2.1 Search methodology

A systematic search was performed by examining PubMed, Cochrane, Embase, Web of Science, and Scopus databases for relevant scientific literature from January 2010 to April 2022. The search was centered around 3 keywords which all articles included: focused ultrasound, glioblastoma, and rodents. For each of these keywords, the search algorithm included synonyms, acronyms, or modalities relevant to the term and controlled vocabulary terms such as Medical Subject Headings (MeSHterms) in PubMed (**Table 1**). The results obtained were filtered neither by publication year nor impact factor of the publishing journal. The electronic systematic review management software, Covidence (Melbourne, Victoria, Australia) was used for full-text review and data extraction, which was then exported to Microsoft Excel V.16 (Redmond, WA, United States).

**Table 1:** Example of search algorithm created for PubMed. [mesh] refer to mesh terms and [tw] refer to keywords.

PubMed: 1,555 search results	
(“High-Intensity Focused Ultrasound/methods”[mesh] OR “focused ultrasound”[mesh] OR “Ultrasonic Therapy”[mesh] OR “Blood-Brain Barrier Opening”[mesh] OR “Focused Ultrasound Therapy”[mesh] OR “Microbubble”[mesh] OR “Immunomodulation”[mesh] OR “sonodynamic therapy”[mesh] OR “radiosensitization”[mesh] OR “thermal ablation”[mesh] OR “HIFU”[tw] OR “high-intensity focused ultrasound”[tw] OR “ultrasound”[tw] OR “blood-brain barrier”[tw] OR “sonodynamic therapy”[tw] OR “thermal ablation”[tw] OR “radiosensitization”[tw] OR “immunomodulation”[tw] OR “focused ultrasound”[tw] OR “FUS”[tw] OR “LIFU”[tw] OR “Low-intensity focused ultrasound”[tw]) AND (“Brain neoplasms/therapy”[mesh] OR “Glioma/therapy”[mesh] OR “brain tumor”[mesh] OR “glioma”[mesh] OR “glioblastoma”[mesh] OR “intracranial tumor”[mesh] OR “brain tumors”[tw] OR “glioblastoma”[tw]) AND (“Rat”[mesh] OR “rat”[tw] OR “Rats, Sprague-Dawley”[mesh] OR “Rats, Wistar”[mesh] OR “Mouse”[mesh] OR “mouse”[tw] OR “rodent”[tw]).	

### 2.2 Inclusion criteria

Using the search algorithm described above, a database of articles was created. Studies involving the use of FUS in a rodent model (rats or mice) with an intracranially-implanted glioblastoma cell line were included. Intracranial implantation was not limited by the site of injection as long as the tumor site was in the brain.

### 2.3 Exclusion criteria

Studies for which full-text access was not readily available were excluded from this review. Studies that were not relevant to the application of FUS on a GBM intracranial animal model were also excluded.

### 2.4 Data extraction and analysis

Study assessment and data extraction were conducted by independent reviewers. The articles were initially screened in Covidence by title and abstract using the inclusion and exclusion criteria. All articles that were accepted by the initial screening underwent full-text review prior to data extraction. Any conflicts between reviewers in this step were resolved by an arbiter.

A data collection form was created for the organized extraction of key data points (**Supplementary Table 1**). Data exported in Microsoft Excel and imported into MATLAB (MathWorks, Natick, MA, United States) and RStudio (Boston, MA, United States) for processing. Data was collected only for *in vivo* portions of each study. For numerical data categories (e.g., frequency, pressure, duty cycle), the mean, median, mode, maximum, minimum, and standard deviation were calculated across each paper. The total number of papers reporting each statistic and the total number of unique data points were recorded. If the publication reported a range of values, either the lower or upper bound was included in the calculations depending on the category (animal model categories: lower bound; FUS parameter categories: upper bound). This was chosen to create consistency for the calculations when ranges of values were given. For qualitative categories (e.g., breed, cell line), the mode, number of reporting papers, and any unique values were recorded. If the author did not specify consistent transducer parameters such as element size, radius of curvature (ROC), focal depth, and focal region width but they were available for reference from other papers in this review or datasheets online, those values were included. For numerical categories, violin plots were created to examine data distributions using the R software package (University of Auckland, Auckland, New Zealand). To determine if parameters were differently chosen for mice and rat models, a two-sample t-test was run for each parameter to determine statistical significance between the two models (p<0.05).

Following data collection, an analysis of collected treatment outcome data were compared. Three measures of treatment outcomes were calculated for each study. First, the survival time was quantified by calculating the percent increase of median survival of the full treatment group (FUS + microbubbles (MB) + therapeutic compound) compared to the monotherapy group (therapeutic compound only). Next, the extent of tumor inhibition when comparing full treatment group to the monotherapy group was calculated by determining the percent change of tumor size between the two groups, such as comparing the respective volumes or bioluminescence of tumors. Similarly, extent of increased drug uptake was calculated by determining the fold change of drug uptake between the full treatment group and the monotherapy group, such as comparing the concentration of that drug in tissues. For each of these outcomes, if the variable was not explicitly stated, where possible the value was estimated based on the graphs provided. Multivariate linear regression methods were used to determine if there were any significant relationships between the continuous independent variables and the treatment outcomes. To account for inconsistency amongst the reported data across studies, only variables that were reported by at least two-thirds of all papers were included in the regression. Additionally, only variables that were not significantly different between mice and rats were included. Analysis of variance was performed to determine success, where p<0.05 indicated a significant regression.

## 3 Results

### 3.1 Study selection

**Figure 3:**
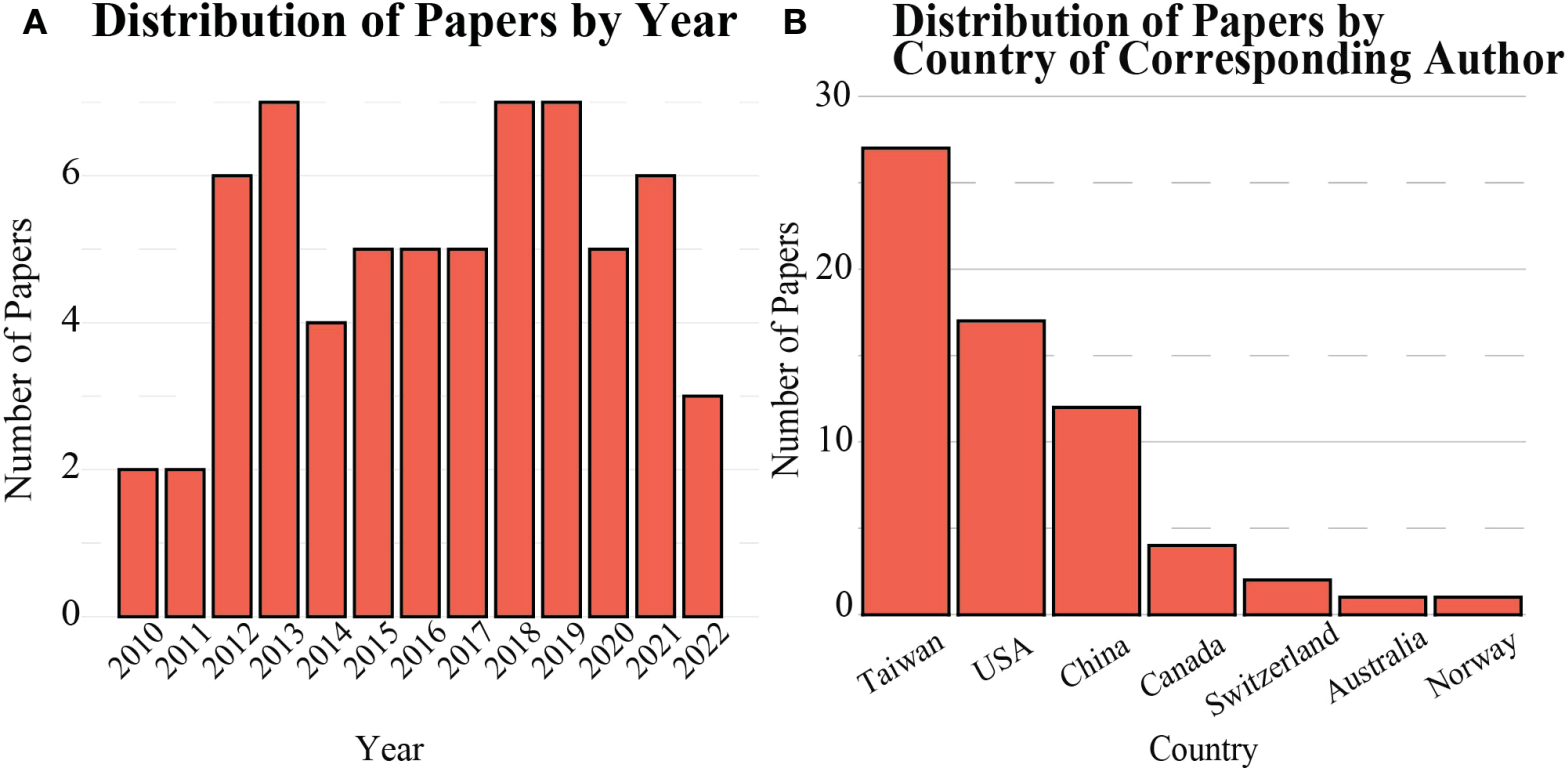
Distribution of scientific literature published on FUS-mediated BBB opening for *in vivo* rodent models **(A)** over time, and **(B)** by country.

In total, 64 publications featuring a FUS-induced BBB opening applied to an *in vivo* intracranial GBM rodent model were identified between January 2010 and April 2022 and were included in this review (**Figure 3A**). The studies were distributed among 33 corresponding authors from 7 countries, primarily in Taiwan, the United States, and China (**Figure 3B**).

### 3.2 Animal models

**Figure 4:**
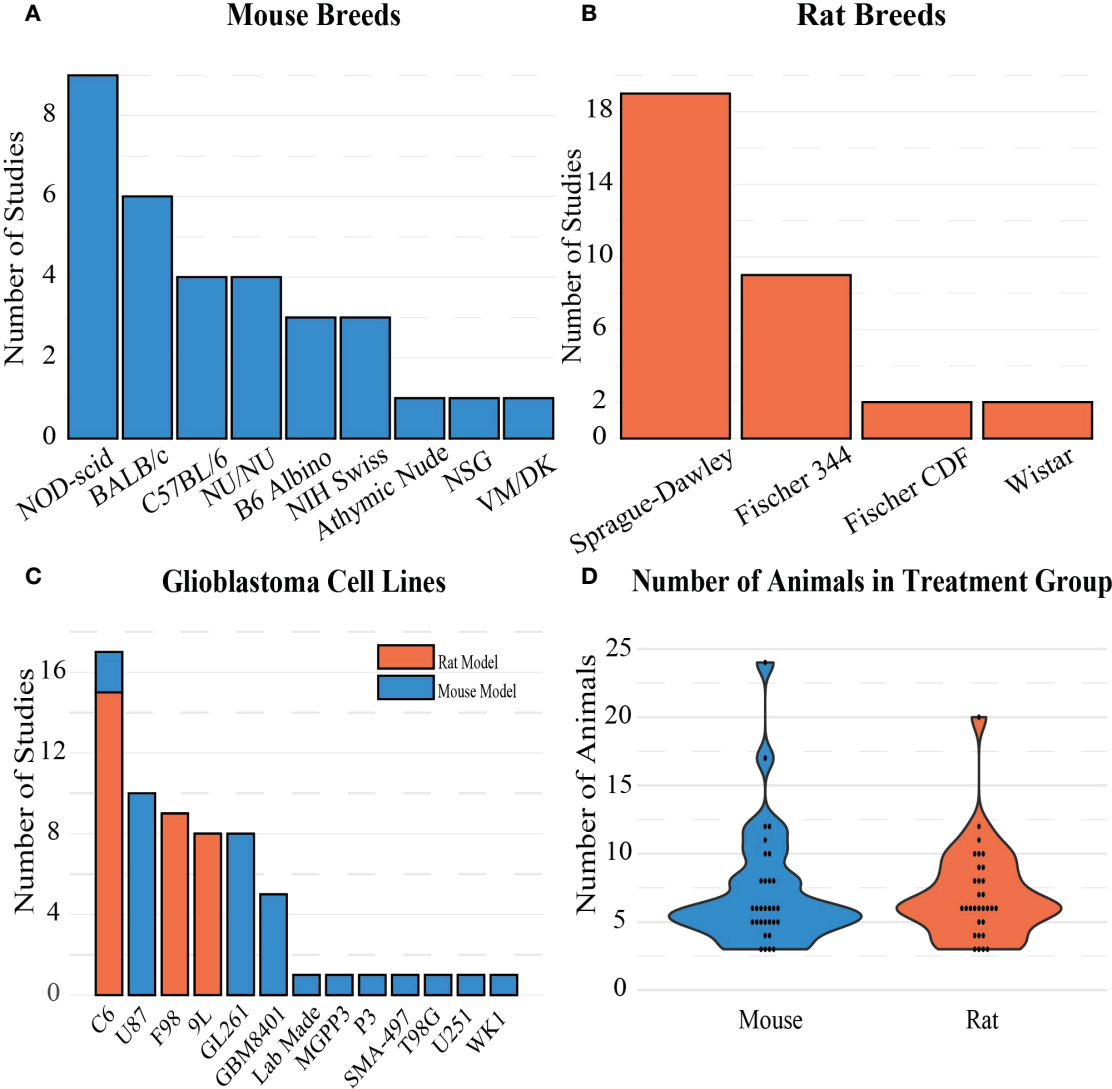
Frequency of **(A)** mouse breeds, **(B)** rat breeds, and **(C)** GBM cell lines in the included studies. **(D)** Distribution of sample sizes chosen for treatment groups in the studies.

Rodent demographics such as type, breed, age, and weight, as well as tumor model information such as cell line, number of cells, and implantation depth into the brain were extracted (**Figure 4**). Of the 64 studies included in this review, 32 used a rat model and 32 used a mouse model. For studies in which rat models were used, the most common breed and cell line used for implantation were Sprague-Dawley (n=19, **Figure 4B**) and C6 gliomas (n=15, **Figure 4C**), respectively. Mouse models were more varied, where the most utilized breed and cell line were NOD-scid (n=9, **Figure 4A**) and GL261 mouse glioma (n=9, **Figure 4C**), respectively. The C6 glioma cell line was the only cell line used in both rats and mice. Although the average number of intracranially-implanted cells was higher per rat than per mouse, the number of cells was highly variable (rats: 2.5e^3^-10e^7^; mice: 5e^2^-10e^6^). Tumor growth and size on the date of the FUS-induced BBB opening procedure were commonly measured using MRI to estimate tumor volume or diameter. Original tumor permeability is dependent on the stage of the tumor, so the age of the tumor at treatment time can impact how easily FUS can increase permeability. On average, the tumor size at the time of treatment for rats was 41.14 ± 46.56 mm^3^, while for mice it was 34.81 ± 59.43 mm^3^, indicating a wide range of tumor sizes at the beginning of treatment for both models. Otherwise, tumor growth was estimated using fluorescent or luminescent biomarker-based imaging techniques. For any *in vivo* study, animal age and weight are also contextually important as they can factor into the animal lifespan and viability when tumors are present. Rat models were implanted with tumors at the average age and weight of 9.11 ± 2.47 weeks and 225 ± 42 g, respectively. Mice were on average implanted with tumors at the age and weight of 6.75 ± 3.85 weeks and 20.75 ± 2.5 g, respectively. These ages correspond to the time in the lifespan in rodents when rodents reach mature adulthood. Finally, it is also worth noting that the implantation depth is important, as it can be more difficult to aim for and treat deeper seated tumors, and the location of tumor growth can have differing neurological effects on the animal. Rats were implanted with tumors at an average depth of 4.33 ± 0.56 mm, while mice were implanted at an average depth of 3.1 ± 0.93 mm. The rat brain is on average 3 times larger than the mouse brain, so there is a discrepancy between the volumes of these two rodent models and the injection depths. Overall in rat models, tumors were implanted more superficially when compared to mouse models. This is most likely because the rat skull is thicker than the mouse skull, making it harder for FUS to penetrate into deeper implantation sites in the brain.

### 3.3 BBB openings

#### 3.3.1 Type of therapeutic compounds used

**Figure 5:**
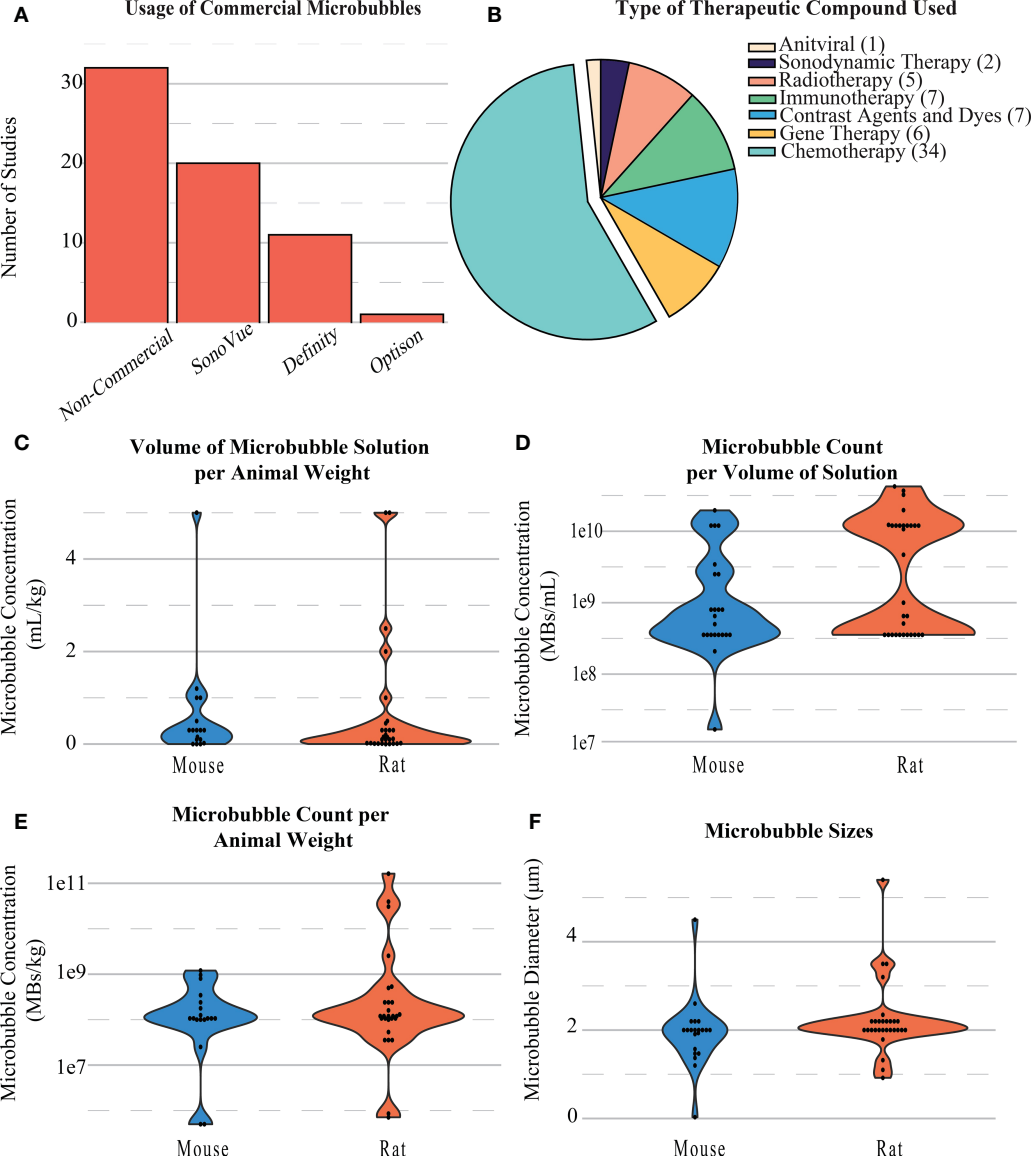
**(A)** Distribution of the types of microbubbles used in BBB opening studies. **(B)** Distribution and type of compounds delivered during BBB openings. **(C)** Distribution of the volumes of microbubble solution per animal weight in BBB opening studies. **(D)** Distribution of the microbubble count per volume of solution injected during BBB openings. **(E)** Distribution of the microbubble count per animal weight during BBB openings. **(F)** Distribution of the diameter of microbubbles during BBB openings.

Each of the 64 studies included in this review was categorized based on the compound delivered to the tumor for treatment. Just over half of the papers (n=34) were focused on delivering a chemotherapeutic drug (most commonly doxorubicin). Other BBB opening studies delivered immunotherapy (n=7), gene therapy (n=6), radiotherapy (n=5), sonodynamic therapy (n=2), and anti-viral drugs (n=1) to GBM tumors. **Table 2** summarizes the types of therapeutic compounds used in these studies, the mode of administration (e.g., free, liposomal) and how successful they were at treating intracranial GBMs. The remaining papers used a non-therapeutic contrast agent or a dye/stain to confirm the BBB opening (**Figure 5B**). The reported molecular weights of the compounds tested ranged from 194 Da (free Temozolomide) to 150 KDa (antibodies).

**Table 2:** Therapeutic compounds tested with FUS-mediated BBB opening *in vivo* for the treatment of GBM.

Drug Name	Drug Category	Size of Free Drug (kDa)	Average Concentration Delivered (mg/kg)	Forms	Fold Increase of Delivery Compared to Monotherapy	Median Survival Percent Increase Compared to Monotherapy	Percent Inhibition Compared to Monotherapy
Doxorubicin (N=15)	Chemotherapy	0.544	5.97 ± 3.67 (N=12)	Free (N=4), Liposomal (N=8), Exosomal (N=1), Nanoparticles (N=2)	1.79 ± 0.92 (N=12)	74.98 ± 62.39% (N=7)	40.94 ± 32.77% (N=6)
Carmustine (N=5)	Chemotherapy	0.214	5.99 ± 4.14	Free (N=1), MB-loadded (N=2), Nanoparticle (N=1), MB+Antibody (N=1)	8.92 ± 8.32	53.09 ± 32.75%	84.85 ± 12.32%
Monoclonal Antibodies (N=5)	Immunotherapy/Antiangiogenic Therapy	150	28.10 ± 19.66 (N=3)	Free (N=3), Conjugated (N=2)	3.03 ± 2.12	48.41 ± 16.59% (N=3)	72.28 ± 12.23% (N=3)
Temozolomide (N=5)	Chemotherapy	0.194	52 ± 32.65	Free (N=3), Liposomal + MGMT Inhibition (N=2)	2.91 ± 3.52 (N=4)	47.49 ± 38.90% (N=4)	56.50 ± 24.77% (N=1)
HSV-TK/Ganciclovir (N=3)	Gene Therapy, Antiviral	0.255 (GCV)	46.67 ± 38.59 (GCV)	DNA-loaded MBs (N=1) ,Adenovirus (N=1); Free GCV (N=3)	2.02 ± 0.12 (N=2)	36.11 ± 2.78%	54.93 ± 35.35%
Boron Carriers (N=3)	Radiotherapy	0.208 (BPA)	375 ± 125 (N=2)	Free (N=2), Loaded in MBs (N=1)	1.75 ± 1.44	N/A	2.46% (N=1)
Cisplatin (N=2)	Chemotherapy	0.301	1.50 ± 1.00	Nanoparticle	1.67 (N=1)	64.86 ± 3.88%	18.52% (N=1)
DVDMS (N=2)	Sonodynamic Therapy	1.23	1.50 ± 0.50	Free, Liposomal	5.32 ± 1.89	98.53% (N=1)	63.44 ± 47.09%
Etoposide (N=2)	Chemotherapy	0.589	12.50 ± 7.50	Free	7.42 ± 0.57	19.79 ± 11.79%	50.66% (N=1)
Paclitaxel (N=2)	Chemotherapy	0.853	6.50 ± 3.5	PLGA Nanoparticle (N=1), Liposomal (N=1)	2.50 ± 0.50	15.22 ± 0.41%	86.28 ± 11.28%
Cabazitaxel (N=1)	Chemotherapy	0.835	10	Nanoparticle	-0.33	-50%	N/A
Carboplatin (N=1)	Chemotherapy	0.371	50	Free	1.90	42.67%	48.21%
Gambogic Acid (N=1)		0.629	N/A	GA-loaded MBs	98.67%	36.36%	N/A
Interleukin-12 (N=1)	Immunotherapy	70	0.0003	Free	1.80	15.38%	84.76%
Irinotecan (N=1)	Chemotherapy	0.587	20	Free	3.64	-8.93%	0%
Short Hairpin RNA (N=1)	Gene Therapy	N/A	10.4	Liposomal	N/A	66.67%	40.74%

N/A, Not applicable.

Across all papers, the majority of microbubbles used were produced in-house (n=30). Commercial microbubbles were used in some studies, namely, SonoVue Bracco, Milan, Italy, n=20), Definity (Lantheus, Billerica, MA, United States, n=11), and Optison (General Electric, Boston, MA, United States, n=1) (**Figure 5A**). MB concentrations were commonly reported as either volume of microbubble solution per weight of the animal (mL/kg, **Figure 5C**), number of microbubbles per weight of the animal (MBs/kg, **Figure 5E**), or number of microbubbles per volume of solution administered (MBs/mL, **Figure 5D**). The size of microbubbles was also reported or inferred if using a commercial formulation. For rat models, the number of microbubbles per volume of solution was 8.73e^9^ ± 1.12e^10^ MBs/mL, concentration per animal weight was 8.84e^9^ ± 3.15e^10^ MBs/kg, and size of MBs used was 2.23 ± 0.81 µm. The number of microbubbles per volume of solution was 3.11e^9^ ± 5.20e^9^ MBs/mL, concentration per animal weight was 2.61e^8^ ± 3.40e^8^ MBs/kg, and size of MBs used 1.92 ± 0.75 µm on average for mice (**Figure 5F**).

#### 3.3.2 Treatment settings

**Figure 6:**
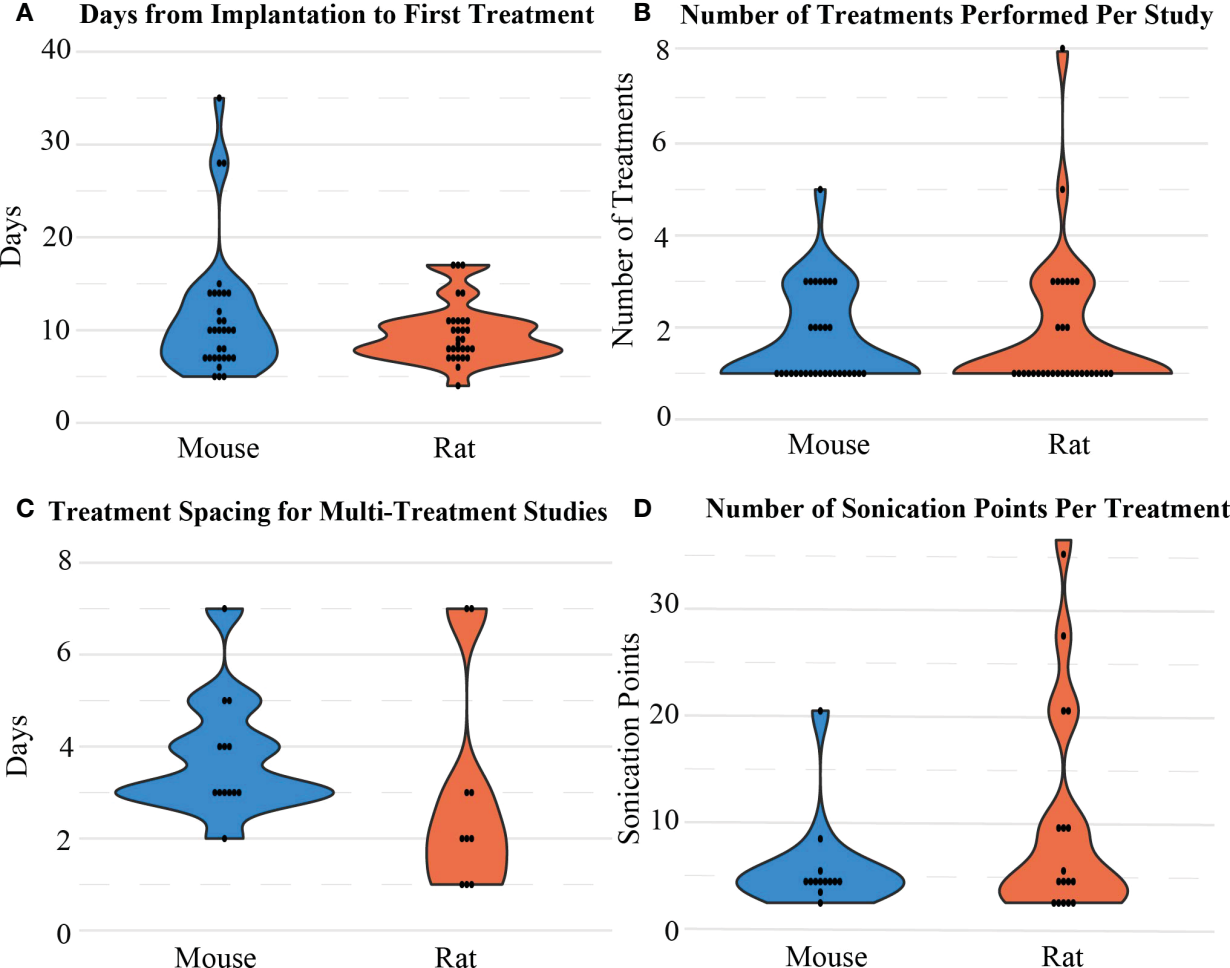
Distribution of **(A)** the time intervals between implantation and the first BBB opening treatment, **(B)** the number of sonication treatments performed, **(C)** the treatment spacing/interval considered between 2 treatment sessions, and **(D)** the number of sonication points considered in one treatment session for mice and rat models.

Most FUS treatments occurred within 1 to 2 weeks post-tumor implantation (n=48, **Figure 6A**). Although many papers only involved one FUS procedure in their treatment paradigm, for the 24 studies that included more than one procedure, most occurred two or three times (3.04 ± 1.31, **Figure 6B**), with an average of 3.39 ± 1.76 days between sessions (**Figure 6C**). During the procedure, almost half of the studies involved sonication at more than one location in the brain, which was often done in a grid pattern or in focal points covering the shape of the tumor when MRI guidance was accessible (**Figure 6D**). An example of a grid pattern can be found in **Figure 1**. The number of sonication points was higher in rats when compared to mice (9.47 ± 9.86 vs. 5.38 ± 4.41, respectively).

#### 3.3.3 FUS parameters

**Figure 7:**
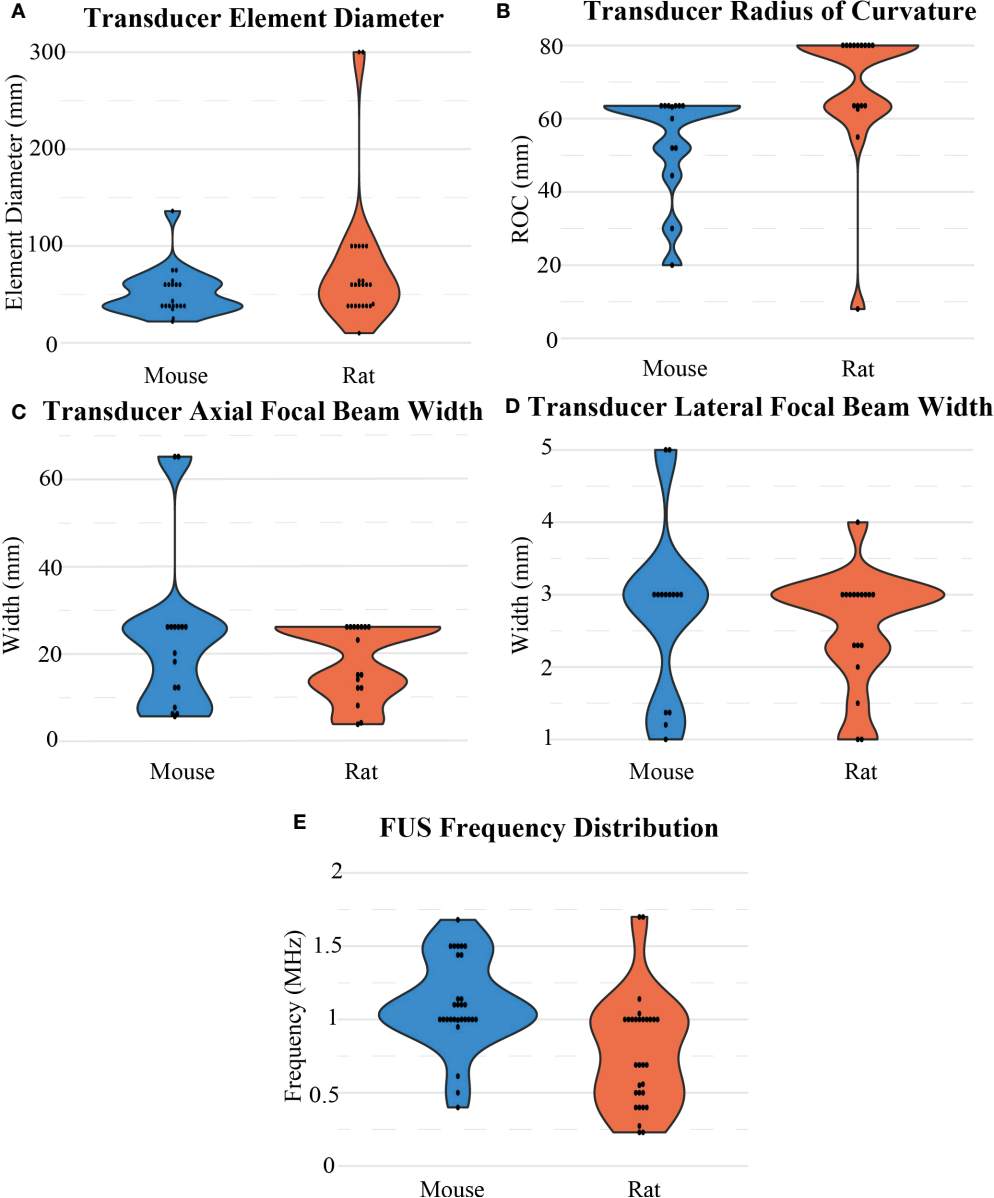
Distribution of FUS transducer properties for BBB opening experiments for mouse and rat models including **(A)** transducer element diameter, **(B)** transducer radius of curvature, **(C)** axial and **(D)** lateral widths of a focal region, and **(E)** FUS frequency.

**Figure 8:**
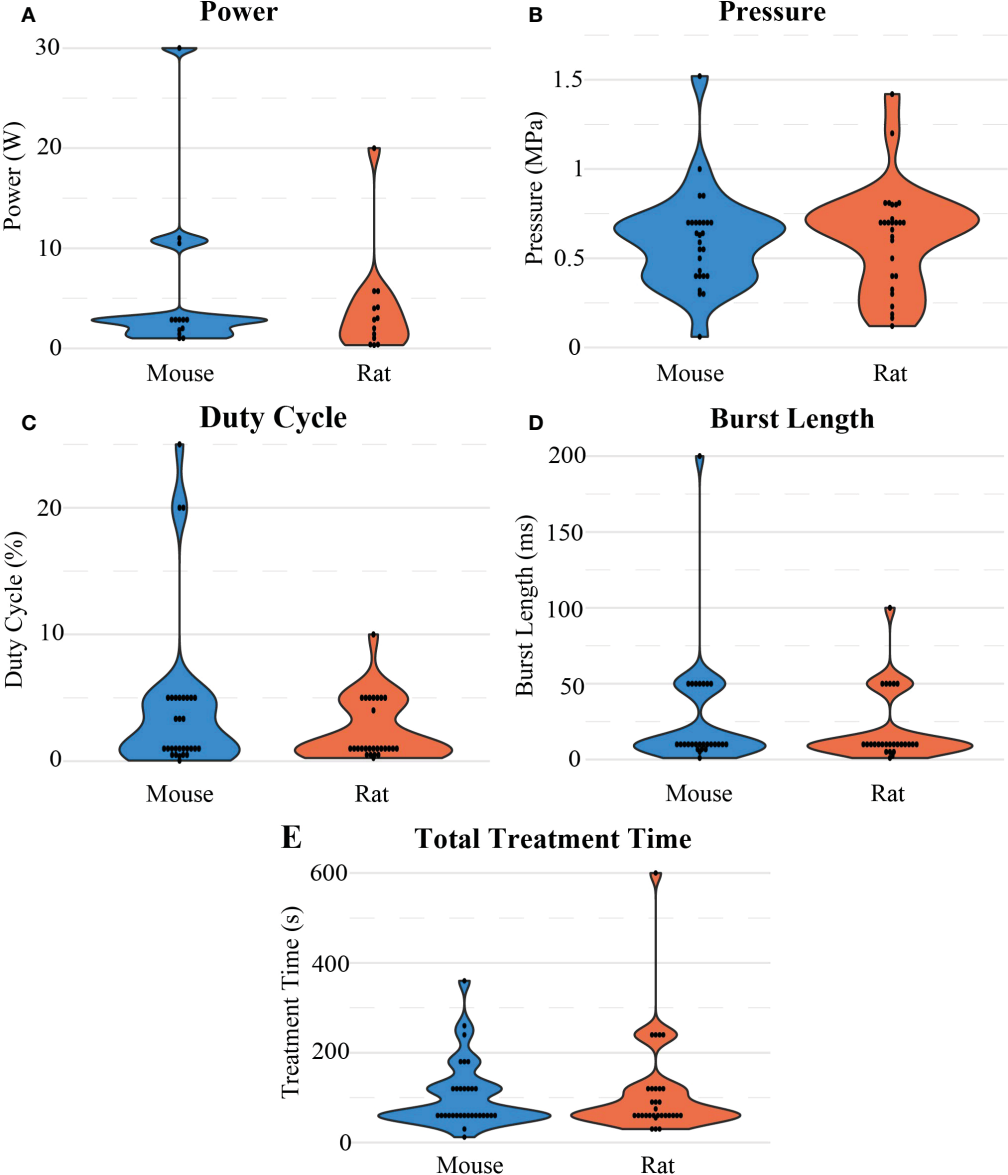
Distribution of different sonication parameters: **(A)** Power, **(B)** pressure, **(C)** duty cycle **(D)** burst length, and **(E)** total treatment time considered in the reviewed studies for FUS-mediated BBB opening experiments in mouse and rat models.

The FUS parameters used in each BBB opening were reported inconsistently. At least 80% of BBB opening studies reported FUS frequency, pressure, total treatment time, burst length, and duty cycle. Besides frequency, there were no other significant differences observed between mice and rats in these categories. For rats, frequency (**Figure 7E**) was on average 0.77 ± 0.37 MHz after outlier removal of a study that used at 10 MHz transducer, while in mice, frequency was on average 1.11 ± 0.29 MHz. For both animal models, 1 MHz was the most commonly used frequency, and but reported values ranged from 0.23 to 2.5 MHz. The resulting pressures (**Figure 8B**) reported by researchers in these studies may differ from the actual *in vivo* pressures that occurred physiologically during a sonication because they were measured in a different environment, such as a free field environment without acoustic reflections. These pressures were on average 0.64 ± 0.32 MPa, ranging from 0.12 to 1.9 MPa. Total treatment time was on average 107.61 ± 93.19 seconds, but was most commonly 60 seconds. Pressure was reported as peak-negative amplitude pressure. One outlier study had a burst length and duty cycle at 1000 ms and 25%, respectively. After removal of this outlier, the average burst interval was 23.29 ± 32.03 ms, while average duty cycle was 2.98% ± 3.90%. Duty cycle and burst length were most commonly 1% (**Figure 8C**) and 10 ms (**Figure 8D**), respectively. As mentioned, there was no significant difference when comparing these values between rats and mice.

#### 3.3.4 FUS devices used

Additionally, pressure and the resulting intensity were based upon other parameters of ultrasound transducers, such as power (**Figure 8A**), element size (aperture diameter; **Figure 7A**) and ROC (**Figure 7B**). With frequency, those parameters create a resulting focal region of high pressure at a depth and focal size (axial and lateral full width at half maximum; **Figures 7C, D**), often characterized by the volume or area where the pressure is at or above half of the maximum pressure. Given these design considerations, its important to choose the right transducer. The tightness of a focal region during a BBB opening is important to consider when treating GBMs, as for some therapies it would be preferable to only target tumor regions while leaving healthy brain tissue unaffected. In a single element transducer, the ROC corresponds to the focal depth, but it can also be reported as the distance between the exit plane of the transducer and the focal region. This can be altered by various methods, such as by using focusing cones or focus region steering in multi-element transducers The geometry of the transducer is not the only important parameter to consider, making it that much more important to report these values for reproducibility. For the 45 papers that reported which company, ultrasound system, and/or transducer type they used, a variety of companies were involved (Panametrics [n=16; Baker-Hughes, Houston, TX, United States], Imasonic [n=10; Voray-sur-l’Ognon, France], Sonic Concepts [n=6; Bothell, WA, United States], and FUS Instruments [n=6; Toronto, ON, Canada]).

For these commonly used transducers, the smallest physical dimensions of the transducers were Panametrics (diameter = 38 mm, ROC = 63.5 mm, frequency = 1 MHz). These parameters corresponded to a focal region that was 26 mm axially and 3 mm laterally. For comparison, the Imasonic transducers used most often had a diameter of 60 mm and a ROC of 80 mm, which across multiple frequencies (0.4, 0.5, and 1.5 MHz) had average focal regions of approximately 15 mm axially and 2.5 mm laterally. As such, the size of the transducer was not indicative itself of how large the focal region would be. Overall, (29) reported using the smallest FUS transducer at 10 mm diameter and 8 mm ROC at 1.7 MHz, corresponding to a focal region of 4 mm axially and 1 mm laterally. Although this study used the smallest transducer included in this analysis, this transducer did not produce the smallest focal region, as (30) used a 10 MHz transducer to create a highly focused region at 3.7 mm axially and 1 mm laterally. On the other end of the spectrum, the 1024-element Exablate Neuro (n=2, Insightec, Tirat Carmel, Israel), designed for clinical use, was also used for *in vivo* rodent GBM experiments, which had an element diameter of 300 mm. The focal region data could be determined for 33 of the 64 papers in this review, and on average, the focal sizes were 20.71 ± 14.01 mm axially and 2.55 ± 1.08 mm laterally. There was no apparent difference in the resulting focal sizes when comparing what devices were used for mice (23.22 ± 17.11 mm axially and 2.54 ± 1.29 mm laterally) or rats (18.04 ± 8.21 mm axially and 2.55 ± 0.78 mm), and the physical transducer diameters were similar (52.43 ± 23.91 mm vs. 59.27 ± 25.50 mm, respectively) after outlier removal. The main difference was in the ROC of the transducers, as the that was larger in rats than in mice (68.72 ± 17.97 mm vs. 56.05 ± 12.47 mm, respectively).

When choosing which system to use for FUS based experiments, beyond the scientific qualities of the device researchers need to consider availability and cost-effectiveness. In the decade that this review covers, new FUS devices became available over time. For example, while the Panametrics and Imasonic transducers were available throughout the entire span of the review, the first reported use of a Sonic Concepts transducer was not until 2015, and likewise, the Canadian-based company, FUS Instruments, was not mentioned until 2020. FUS devices require a power source, amplifiers, and a function generator to function. Therefore, these systems can cost tens of thousands of dollars to operate, especially as they get more technically advanced. Researchers may be limited to a single setup, which could limit experimentation capabilities.

#### 3.3.5 Treatment outcomes

The final information collected for this review was treatment outcomes to determine whether opening the BBB permitted successful treatment of GBMs. When determining whether a BBB opening treatment regimen was successful, the studies included reported 4 main outcomes: BBB permeability (n=34), drug uptake in GBM tumors (n=54), effect on tumor growth (n=37), and effect on animal survival (n=33). To assess BBB permeability, papers commonly used a dye such as Evan’s Blue (n=26) or Trypan Blue (n=2). Although almost every paper underwent contrast-enhanced MRI, some papers assessed BBB permeability by reporting relaxation rates (R1, n=5; R2, n=1) or volume transport rates (Ktrans, n=2) of gadolinium. Drug uptakewas measured in several ways, but most commonly involved tissue extraction and then subsequent measurement *via* methods such as liquid chromatography (n=17), mass spectrometry (n=11), fluorometric assays (n=7) and ELISA (n=5). Otherwise, drug uptake was also measured using *ex vivo* whole organ fluorescence (n=12). Some studies measured samples for radio-labeled drugs uptake using PET or Micro-SPECT/CT scans (n=8). Tumor growth was commonly measured for volume and area using MRI (n=26) or *via* other noninvasive methods such as bioluminescence imaging with luciferase-tagged tumor cells (n=9). Another commonly reported outcome was body weight (n=10). Passive cavitation detection or other methods to measure sub-harmonic and broadband signals were also employed during treatments (n=7), which were used to allow for closed-loop pressure control. This refers to the monitoring of reflected soundwaves where the presence of subharmonic, harmonic, and ultraharmonic frequencies or broadband noise (non-harmonic frequencies) indicates potential microbubble cavitation (31). Finally, hematoxylin and eosin (H&E, n=46) and TUNEL (n=11) were commonly used histology stains performed for the detection of pathologic or apoptotic tissue. Although the treatment outcomes were reported differently across studies, the results were generally positive with significantly improved outcomes compared to control groups in all but 5 of the studies included.

Across all studies, the addition of FUS and MBs to various therapies increased median survival time by 46.52 ± 42.31%, inhibited tumors by a percentage of 59.71 ± 33.01%, and increased uptake of therapeutic molecules across the BBB by 3.18 ± 3.90 fold when compared to the monotherapy being tested against. When comparing which animal model had better successes in treating GBMs, there was no significant difference across all three categories (p=0.32, p=0.39, p=0.35 respectively). There were only weak correlations between the extent of increased therapeutic uptake due to BBB opening to the outcomes of treatment success (i.e., tumor inhibition r(25)=0.29, p=0.14; median survival r(24)=0.29, p=0.15).

Next, the treatment outcomes were compared to the independent variables reported in this review to determine if there were any indicators of treatment successes *via* a multivariate linear regression. Only variables that were reported on by two-thirds of the studies (N=42) were included. The parameters that were included in this regression included the concentration of microbubbles in both volume and number of microbubbles per animal weight, microbubble size, the time from implantation to treatment, the number of treatments, FUS frequency, pressure, burst length, treatment time, and pulse repetition frequency (PRF). The overall regression for median survival time was not statistically significant (R^2^ = 0.49, F(10, 4) = 0.38, p = 0.9). Similarly, the overall regression for FUS parameters on improving tumor size was not significant (R^2^ = 0.63, F(36, 10) = 1.36, p = 0.34). Treatment date from implantation (p=0.06) and microbubble size (p=0.09) most closely predicted tumor size. Finally, the regression model for difference in drug uptake due to FUS was statistically significant (R^2^ = 0.78, F(10, 16) = 5.76, p = 0.001). Microbubble concentration (MBs/kg, p = 0.04), microbubble size (p = 0.002), and pulse repetition frequency (p = 0.0001) were significant predictors of improved drug delivery due to FUS.

## 4 Discussion

This review has described the experimental design of several studies investigating the FUS parameters for BBB permeability in various types of rodent GBM models and compounds tested. The studies described in this review feature some common parameters that may guide future BBB permeability studies. Due to the high number of of different variables that go into performing these studies and the inconsistency in reporting of variables across studies, it was difficult to make conclusions about which parameters were most important for favorable results. Overall, microbubble characteristics such as size and concentration seemed to be indicators of success, above traditional indicators such as pressure and frequency. Additionally, it is important to note that histological findings were not taken into account when analyzing treatment outcomes, so a future analysis that looks at safety of these outcomes might be warranted.

### 4.1 Common BBB-opening study setup

When establishing the tumor model and treatment plan, the initial treatment was most commonly performed 7- to 14-days following tumor implantation for both rats and mice, although this was dependent on the cell growth rate and number of cells implanted (**Figure 6A**). Furthermore, selection of study parameters such as the number of treatments or sonication location was dependent on drug perfusion and the therapeutic potency of the drug being delivered. One-third of the studies included in this review performed more than one FUS treatment with, on average, approximately a 3-day interval (e.g., days 7, 10, 13) between each treatment (**Figure 6C**). The effects of BBB openings are transient, and it has been reported that repeated BBB openings do not appear to cause long term BBB damage (32). Almost half the studies reported FUS treatment at multiple sonication point during a singular treatment, which increased the area of potential perfusion (**Figure 6D**).

### 4.2 Microbubbles and dosage

The effectiveness of the BBB opening is dependent on the mechanical cavitation of the microbubbles; therefore, dosage and size of microbubbles is an important variable for efficiency and safety. Commonly used microbubbles, such as SonoVue and Definity (**Figure 5A**), can be delivered intravenously before treatment at a dose greater than 0.01 mL/kg of animal weight for efficient BBB opening without the presence of unstable cavitation. SonoVue and Definity have a concentration of 3.55e^8^ and 1.2e^10^ microbubbles/mL, respectively, and sizes of 2 and 2.2 µm, respectively. This corresponds to microbubble weight concentrations of 3.55e^6^ and 1.2e^8^, respectively.

Besides the commercial microbubbles, a number of studies created their own formulations of micro bubbles that each have different concentrations and sizes, which can have an impact on the outcome of a BBB opening. Formulations of microbubbles from the various studies are detailed in **Supplementary Table 1**. Polyethylene glycol (PEG) was sometimes coated on the surface of microbubble formulations to provide stability, solubility, and bio-compatibility to microbubbles, allowing immune escape and allowing the microbubbles to circulate throughout the body (33). PEG was most commonly conjugated to 1, 2-Distearoyl-sn-glycero-3-phosphoethanolamine (DSPE) in the shell of the microbubble. Besides DSPE, Other lipids commonly present in the microbubble shell included 1,2-dipalmitoyl-sn glycero-3-phosphocholine (DPPC) and 1,2-distearoyl-sn-glycero-3-phosphoglycerol (DSPG). Because of the amphipathic nature of these phospholipids, they naturally form into stable microbubbles (34). Although on average these manual microbubble formations had similar sizes to SonoVue and Definity, the manufacturing processes may lead to higher size variability when comparing commercial to non-commercial microbubbles. Besides the dosage and average size of microbubbles, it has been previously shown that microbubble variability can contribute to the extent of the BBB opening (35). Therefore, instead of microbubble size or concentration, some scientists are recommending that total gas volume of microbubbles may be a better indicator of BBB opening (36). Even so, microbubbles seemed to play an important role in the success of improved drug perfusion, consistent with findings of other studies (36, 37).

### 4.3 Choice of FUS research system

When deciding which FUS instrumentation to use in rodents, more than one transducer element was not required for minimal attenuation of the ultrasound signal in the rodents’ thin skull. However, element size (**Figure 7A**) and ROC (**Figure 7B**), which are important in determining the focal point geometry, need to be appropriate for the animal model and desired size of the BBB opening. As the tumor size is typically a few millimeters in diameter before treatment, having a transducer with a focal region small enough to cover the tumor without covering any surrounding tissue may be important, depending on the drug being delivered. FUS frequency also correlates to focal region size, with higher frequencies resulting in a smaller focal region. Using a FUS transducer with a frequency over 1 MHz does not appear to be necessary to achieve localized BBB opening (**Figure 7E**). It was also shown that the size of the transducer and the resulting focal regions were not discriminated based upon the animal model used. The main difference was in the ROC, which was larger for rats with larger head sizes.

### 4.4 Acoustic pressure

Given the literature collected in this review, it is clear that BBB openings using FUS in conjunction with microbubbles for the treatment of GBM is an established procedure with robust *in vivo* results. However, there are still discrepancies among various researchers regarding parameters used to achieve similar effects. One of the most important parameters when determining the effect of FUS is acoustic pressure. There is a balance between achieving a pressure sufficient to produce an effect while maintaining thermal and mechanical safety. At the peak pressures necessary for BBB opening, it is unlikely that thermal effects would be involved. If those upper limits are exceeded, unstable cavitation can lead to tissue damage (38). For a robust BBB opening, the pressure should be in the range of 0.6 to 0.8 MPa (**Figure 8B**). At this pressure, a 1% duty cycle (**Figure 8C**) with a burst length of 10 ms (**Figure 8D**) for 1 minute (**Figure 8E**) is recommended for significant therapeutic outcomes. Some studies were able to achieve desired pressure by passive cavitation detection (e.g., monitoring acoustic backscatter with a passive cavitation detector transducer and increasing the pressure until a cavitation effect was detected) where the pressure was then decreased to a safe value (39–41). **Table 3** shows recommended parameter values for FUS-induced BBB openings.

**Table 3:** Most Commonly Used Parameters for BBB Openings. *Per Sonication Spot.

Parameter	Recommended Value
**Days between tumor implantation and treatment**	7-14 days
**Microbubble Dose**	0.1 mL/kg
**FUS Frequency**	1 MHz
**Power**	1-3 W
**Pressure**	0.6-0.8 MPa
**Burst Length**	10 ms
Total Treatment time*	1-2 min
**Duty Cycle**	1%

BBB openings were achieved with pressures as low as 0.12 MPa (**Figure 8B**) which resulted in either the successful deposition of a target drug into tumor tissue as desired or the release of tumor markers into the bloodstream (42). It is important to note that the pressures reported in the studies included in this review are not all directly measured *in vivo*. For example, (43) used a water tank filled with degassed and deionized water and hydrophone to measure pressure of the output of the transducer in a controlled environment. Acoustic attenuation—especially due to the skull—may lead to pressures that vary from the original measurement and accentuate the need for accurate reporting of FUS parameters for context. Numerous parameters need to be considered before the pressure reported in a study can be replicated. Pressure is a function of the frequency, power, and geometry of the FUS transducer, as well as the acoustic properties of the environment. Higher frequencies are attenuated at a faster rate, so although they can provide tighter focal regions, the distance that the FUS beam has to travel through tissue may dictate what frequencies can be used. This is especially important when considering how far the FUS beam must travel through the skull, which has a very high rate of acoustic attenuation due to wave reflection and absorption compared to soft tissue (44). We found that researchers tended to use higher frequencies for mice when compared to rats, as the skull thickness and overall size of the mouse is smaller than rats. Ultrasound power is transducer-specific but is mainly determined by the intensity needs at the focal region and the surface area of the transducer. The focal region of a FUS transducer is often characterized by the distance from the center, where the intensity or pressure is at half maximum, where transducers with larger diameters and ROC typically have larger focal regions. Given the wide range of transducer dimensions and frequencies used in this review, some BBB openings were more focused than others. Due to the cost of a single FUS transducer, it is likely that many researchers had access to a single instrument, which dictated the parameters used. Access to experimental FUS devices is a limiting factor for research and optimization. Another limiting factor is that this type of research is costly and time-consuming, which is reflected in the low number of animals included in each testing group. On average, studies used 7 animals per testing group, which may not confer to adequate statistical power calculations (**Figure 4D**). This is not to discount the methodology used for these studies, as it is clear that BBB openings using FUS are reproducible. However larger sample sizes may be necessary to ensure that the compounds tested are reliably treating GBM after BBB opening for translational purposes.

### 4.5 FUS treatment dosage

FUS burst length (**Figure 8D**), treatment time (**Figure 8E**), and PRF also varied dramatically among studies. These parameters are important when considering the total energy dose delivered and clinical translation. In this review, it was indicated that PRF, or how short the time between FUS bursts were being delivered, seemed to play a major part in the success of improving drug delivery. Faster pulse frequencies indicate that there are higher rates of shear waves being applied to the BBB. Even so, for the treatment of any disease, much less one affecting the CNS, it is ideal to minimize the total amount of treatment time for patient safety, compliance, and comfort. There is evidence that the threshold pressure necessary to achieve BBB openings is correlated with burst length, and there is also evidence that longer burst lengths as well as higher pulse repetition frequencies can lead to inertial cavitation (45, 46). Therefore, to prevent this cavitation, shorter burst lengths would make treatments safer, but at the expense of needing high pressures to achieve BBB opening. Thermal dose at the parameters used here are negligible. For *in vivo* research on rodents with intracranial GBMs, burst lengths commonly appeared in the milliseconds range, while total treatment periods generally lasted a couple of minutes. Across studies, there was no apparent relationship between burst length or total treatment time and the pressure used in the study, suggesting that minimal changes in these values across these studies do not seem to impact the ability of FUS-induced BBB openings. This gap in the literature demands studies such as this present work, and other comparative and quantitative studies.

### 4.6 Therapeutic compounds tested

Chemotherapeutic drugs, such as doxorubicin and temozolomide, which are already commonly used for GBM treatment, were the most highly in BBB-opening studies (**Figure 5B**). The most commonly tested treatment was doxorubicin, appearing both in liposomal formations and as a free drug. This is notable given that it is not currently approved by the FDA for the treatment of brain tumors. Doxorubicin is one of the most effective chemotherapy agents for soft tissue tumors, but its molecular weight is not favorable for crossing the BBB (47). Therefore, opening the BBB would give this treatment new potential for treating GBMs.

Immunotherapy was the next most commonly tested treatment. Immunotherapeutic drugs consist of high-molecular–weight proteins, such as monoclonal antibodies and interleukins, and without BBB opening would not be able to have access to tumors. Similarly, Bevacizumab, an antiangiogenic monoclonal antibody already approved for brain tumors, works by binding to vascular endothelial growth factor and has been shown to reduce tumor vascularization. The clinical functionality of bevacizumab is not dependent on crossing the BBB to work therapeutically. With a 70 kDa molecular weight, it cannot enter the tumor, which may hinder its efficacy. (47) showed that opening the BBB and subsequent administration of bevacizumab gave the monoclonal antibody unprecedented access to the tumor, resulting in significant decreases in tumor volume and growth rate compared to controls.

FUS-mediated BBB openings also would allow for the use of nanoparticles as drug carriers for enhanced perfusion and release in GBM tumors. Encapsulating drugs in nanoparticles can allow for drug delivery to the tumor in a more efficient manner, requiring less of the drug to achieve the same therapeutic effect. Many of the studies included in this review involved the use of nanocarriers including liposomes (41, 48–51), exosomes (52), and other types of degradable polymer nanoparticles such as polyethylene glycol-based nanoparticles (53, 54). The collective results from the papers utilizing this technology showed that an increase in the permeability of the BBB allowed for greater perfusionof nanoparticles carrying therapeutic payload. Some studies used their nanoformulation for dual purposes, such as using microbubbles as the actual drug carriers. (54) synthesized ^10^boron-containing microbubbles, which were released into the tumor selectively following BBB disruption and microbubble cavitation, allowing for increased distribution of nanoparticles in the GBM tumor. This dual and triple combination delivery with FUS-mediated BBB openings allows for novel treatments options for the treatment of GBMs.

## 5 Conclusion

FUS-mediated BBB opening using microbubbles is a safe, and noninvasive way to treat GBMs using therapies that cannot otherwise cross the BBB. This is evidenced by multiple *in vivo* rodent studies that test the ability of compounds, including chemotherapeutics and immunotherapeutics, to treat intracranial GBMs after the BBB is opened. Studies included in this review inconsistently reported various parameters which could make it difficult to replicate them individually. Even so, these studies overwhelmingly show that across multiple different types of therapeutic compounds, this methodology works to improve treatment outcomes when applying FUS. By compiling these relevant studies and detailing the commonly used, and successful parameters, this review aims to give retrospective insight into successful FUS mediated BBB openings, what researchers are reporting in their literature, and the current gaps in knowledge in the field. Based on the findings from this review, it appears that a FUS transducer operating at 1 MHz, leading to a pressure of 0.6 MPa, with a duty cycle of 1% and a burst length of 10 ms over the course of a 1 minute should lead to a robust BBB opening in a rodent GBM model. Even so, there is still a need to perform comprehensive parameter diagnostics to determine optimal BBB opening specifications. The guidelines in this review aim to pave the path such that the BBB does not remain a limiting factor in the therapeutic treatment regimen for GBM.

## Data availability statement

The original contributions presented in the study are included in the article/**Supplementary Material**. Further inquiries can be directed to the corresponding authors.

## Author contributions

RT, GM, and SK created search algorithms and collected articles. RT and GM followed PRISMA guidelines for final article selection, and SK functioned as an arbiter. RT and GM extracted data, provided data analysis, wrote the manuscript, and created the figures and tables. AH provided guidance for starting the review and collecting articles. AM, BT, and KKL provided critical edits. MA and TK provided inspiration for this review. All authors contributed to the article and approved the submitted version.
